# Supplementary material for: Reversibility in the Physical Properties of Agarose Gels following an Exchange in Solvent and Non-Solvent
Source: Polymers (Basel). 2024 Mar 14;16(6):811. doi: 10.3390/polym16060811 (PMC10975127; doi:10.3390/polym16060811)
Supplement: Supplementary file 1 [file polymers-16-00811-s001.zip › polymers-2903759-supplementary.pdf]

Supplementary Information

Chemical shifts identification for carbon and proton in agarose were given in reference [2]. Those data were obtained from agarose with a degree of methylation 0.24. For Genetics sample with methylation 0.04, the methylated galactose unit (G”) is not identified due to a lack of sensitivity except in proton NMR for the $-CH_3$ signal. Here G is the anhydro-L-galactose and G’ is the D-galactose unit.

Table S1. Chemical shifts of proton and carbon signals for agarose in D_2O at 80°C.

	C1	C2	C3	C4	C5	C6	CH_3
G′	102.63	70.38	82.33	68.93	75.51	61.56	
G″	102.63	70.38	82.33	68.93	75.51	71.89	59.2
G	98.52	70.05	80.31	77.6	75.7	69.64	
	**H1**	**H2**	**H3**	**H4**	**H5**	**H6**	**CH_3**
G′	4.58	3.65	3.78	4.14	3.73	3.80	
G″	4.58	3.65	3.78	4.14	3.73	3.70	3.43
G	5.16	4.13	4.54	4.68	4.57	4.22[a]-4.04[b]	

Table S2. Chemical shifts of proton and carbon signals for agarose in DMSO-*d6* at 80°C.

	C1	C2	C3	C4	C5	C6	CH_3
G′	102.5	70.13	81.35	68.11	75.54	60.8	
G″	102.5	70.13	80.99	68.63	73.52	71.96	58.9
G	97.54	70.37	80.23	76.53	75.12	68.9	
	**H1**	**H2**	**H3**	**H4**	**H5**	**H6**	**CH_3**
G′	4.31	3.45	3.54	3.83	3.44	3.55	
G″	4.31	3.45	3.47	3.76	3.63	3.54[a]-3.46[b]	3.3
G	5.09	3.83	4.22	4.54	4.35	3.91	
